# Cocaine in Hay Fever

**Published:** 1885-12

**Authors:** Mark H. O’Daniel

**Affiliations:** Assistant Physician to the Georgia Lunatic Asylum, Milledgeville, Ga.


### COCAINE IN HAY FEVER.

BY MARK H. O'DANIEL, M. D.,

ASSISTANT PHYSICIAN TO THE GEORGIA LUNATIC ASYLUM, MILLEDGEVILLE, GA.

On September 19th last my attention was directed to the efficacy of cocaine in hay fever by an article with reports of two cases published in the *Medical Record*, from the pen of Dr. G. H. Simmons, of Lincoln, Neb. Having under observation at the time a gentleman who was suffering from the distressing malady, I at once called his attention to it, and recommended its use as prescribed by Dr. Simmons, which was the application of a two per cent. solution to each nostril, and well back into the posterior nares, together with two or three minims dropped into each eye.

The gentleman gave every evidence of great suffering; stated that he had been annoyed with the trouble for nine years. The attacks generally come on the 26th of August and last six weeks. His eyes were congested. He complained of dimness of sight, great fullness in the frontal portion of his head, attended with pain, feeling of constriction across the brow, shortness of breath exhaustion. Every three or four hours he would have attacks and of sneezing, which lasted for a few minutes, followed with profuse flow from the nostrils, which continued for about an hour, wetting as many as three or four handkerchiefs. He has resorted to almost every means, save change of climate, without relief.

The first application afforded him speedy and perfect relief, with the exception of the shortness of breath and exhaustion, both of which were very greatly ameliorated. After having continued the treatuent for a week, he was entirely free from all symptoms.

Now, whether or not the attack was cut short, or the disease had run its course, I am unable to say, but am strongly of the opinion it was due to the cocaine. If the latter, it was of much shorter duration than ever before. While I have no evidence, still I think the use of the remedy timely employed would cut short an attack in its incipiency, and relieve those subject to the disease of much annoyance and suffering.

From my observation of this single case, I must say I esteem it first of all other remedial agents in hay fever, and believe that in the newly-discovered anæsthetic we have a means of relief for patients at home, without having them run off to the mountains. I am delighted to have read Dr. Simmons' article on the subject, and cheerfully recommend its further use to other members of the profession, feeling that they, too, will experience the same happy result.

